# Clinical Comparison of Diode Laser Assisted “v-Shape Frenectomy” and Conventional Surgical Method as Treatment of Ankyloglossia

**DOI:** 10.3390/healthcare10010089

**Published:** 2022-01-04

**Authors:** Sileno Tancredi, Paolo De Angelis, Mario Marra, Michele Antonio Lopez, Paolo Francesco Manicone, Pier Carmine Passarelli, Antonino Romeo, Roberta Grassi, Antonio D’Addona

**Affiliations:** 1 Department of Head and Neck, Division of Oral Surgery and Implantology, Institute of Clinical Dentistry, Fondazione Policlinico Universitario Gemelli, Università Cattolica del Sacro Cuore, 00168 Rome, Italy; studiodentisticotancredi@gmail.com (S.T.); dr.paolodeangelis@gmail.com (P.D.A.); paolofrancesco.manicone@unicatt.it (P.F.M.); piercarmine.passarelli@unicatt.it (P.C.P.); antonixromeo@gmail.com (A.R.); antonio.daddona@unicatt.it (A.D.); 2 ENT, Campus Bio-Medico University, 00128 Rome, Italy; micheleantonio.lopez@gmail.com; 3 Department of Oral Surgery, Tor Vergata University, 00133 Rome, Italy; roberta.grassi@students.uniroma2.eu

**Keywords:** frenectomy, laser, surgery, ankyloglossia

## Abstract

(1) Background: Ankyloglossia, or tongue-tie is a condition, in which the tip of tongue cannot protrude beyond the lower incisor teeth because of short frenulum linguae, often containing scar tissue. Limitations of movement are the most important clinical symptoms of this condition, together with feeding, speech, and mechanical problems. (2) Methods: the present study included two groups of patients (group A and group B) including, respectively, 29 and 32 patients (61 patients total), aged from 8 to 12 and presenting ankyloglossia classified according to the Kotlow’s classification. The patients in group A underwent a common surgical procedure. For the patients of group B, a diode laser device (K2 mobile laser, Dentium, Korea) with a micro-pulsed wavelength of 980 ± 10 nm and power of 1.2 watts was used. The post-surgical discomfort of the patients (recording the pain perceived immediately after the end of the anesthesia and during the following week, using the Numeric Rating Scale (NRS) system) and healing characteristics (recorded using the Early Wound Healing Score or EHS) were evaluated. (3) Results: The results shows that the pain in the patients who underwent laser-assisted frenectomy is significantly reduced (*p* < 0.001) when compared to those who underwent conventional surgical frenectomy, both immediately after surgery (with a reduction in the average NRS of 80.6%) and after the first week (with a reduction in the average NRS of 86.58%). Additionally, in the same patients, an augmentation in the average value of the EHS of 45% was recorded, highlighting significantly (*p* < 0.001) better quality in the healing of the wound within the 24 h after surgery. Moreover, other advantages observed in the use of laser assisted-frenectomy are the absence of bleeding and, consequently, a clear operative field; no need to use sutures; no need to take painkillers or antibiotics after surgery; and having a faster recovery and less time needed to perform the operation. (4) Conclusions: within the limits of the present study, it seems possible to assert that the laser frenectomy performed using the v-shape technique presents a series of advantages if compared to the conventional surgical method.

## 1. Introduction

The orofacial district plays a particularly important role, being involved both in carrying out the basic physiological functions (chewing, speaking, and swallowing) and in the perception of oneself, which plays a crucial role in the ability to relate to others [1]. Aesthetic–functional problems can lead the patient to feel inadequate and to encounter important social problems [1]. In particular, the discomforts caused by alterations of the lingual frenulum are particularly felt by the patients who present them. The lingual frenulum is a little fibro-mucous plica that goes along the midline of the underside of the tongue. It is responsible for the sagittal connection underside of the tongue with the mucosa of the mouth floor, and it has a crucial role in the stability and limitation of the tongue movement [1]. Of the two junctions that it presents (alveolar and lingual), the first one can be placed close to the free gum (defined “marginal”); close to the apex of the tooth root (defined “apical”) or placed below the apex of the tooth (defined “subapical”). Physiologically, the lingual junction is at least (but usually more than) 2 cm distant from the tip of the tongue. Under this distance, it is possible to define a pathological condition known as ankyloglossia. Ankyloglossia or tongue-tie is a condition in which the tip of tongue cannot be protruded beyond the lower incisor teeth because of short frenulum linguae, often containing scar tissue [2]. Limitations of movement are one of the most important clinical symptoms of this condition [3], but it may also presents feeding problems and limitation of the capacity of the newborn to properly assuming breast milk [4,5,6,7,8,9]; orthodontic disorders (related both to the proper bone development and/or the correct positioning of the teeth [7,10]); periodontal implications related to gingival recession [7]; phonatory [7,8,10,11,12,13], mechanical, and swallowing problems [5,7,8,10,12,13,14]; particularly, a short-lingual frenulum limits upward movement such that during deglutition the tongue thrusts anteriorly instead of upward the hard palate, reducing palatal width [15]. It is also associated with the narrowing of the maxillary dental width and the elongation of the soft palate [16], with a prominent risk factor for upper airway collapsibility [17]. Moreover, as said before, in adult patients this pathology can cause severe social problems and important functional limitations [1]. This condition is observed in approximately 3–4% of infants [18,19] but not all infants with tongue-tie have breastfeeding difficulties with incidence varying from 25% to 44% [20,21], but nipple pain in mothers is observed with a prevalence that reaches at 84% [22]. According to Kotlow’s classification [23], there are four classes identifying ankyloglossia, based on the distance of insertion of the lingual frenulum on the tip of the tongue: class I (mild ankyloglossia, 12–16 mm); class II (moderate ankyloglossia, 8–11mm); class III (severe ankyloglossia, 3–7 mm); and class IV (complete ankyloglossia, <3mm). The state of the art for the resolution of this pathology is to surgically intervene on the lingual frenulum. Depending on the gravity of the case (according to Kotlow’s classification) two procedures are used: in case of Kotlow’s class I or II (mild or moderate ankyloglossia) it is performed the frenulotomia, which consist in the cutting of the frenulum; in case of Kotlow’s class III and IV (severe or complete ankyloglossia) it is performed frenectomy, which consists in the surgical removal of the frenulum. All the patients’ conditions that could be interested by this pathology has to be considered [24,25,26,27,28]. In the last decades, clinicians have made considerable efforts in the search for new procedures that could overcome these and other problems, related specifically to oral surgery but also to surgery in other districts [29,30,31]. Among these, excellent results have been achieved with the use of the laser method, and in particular the latest generation diode lasers has shown an excellence in results achieved superior to previous CO_2_, neodymium, and erbium models [32]. In fact, the interaction that the diode laser has with the soft tissues has been proved to be ideal for the use in oral surgery; so much that, according to several studies, it could successfully replace the traditional surgical techniques [33,34]. From then on, clinicians have begun to perform and study the results obtained at first with laser-assisted frenectomy, and then with full laser frenectomy [35]. The aim of this study was to find out if there are any differences, regarding the patient’s related outcomes, such as the discomfort of the patients, and the healing between the laser frenectomy and the frenectomy performed with the conventional surgical method in the intra- and postsurgical phases.

## 2. Materials and Methods

### 2.1. Patient Selection

The present retrospective study included a population of sixty-one subjects (32 females and 29 males) presenting ankyloglossia classified as class III or IV according to Kotlow’s classification, aged from 8 to 12 years (presenting an average age of 9.8 years). The patients of the group A (15 females, 14 males; 16 Kotlow’s class III, 13 Kotlow’s class IV) had undergone a conventional surgical operation of frenectomy, while the patients of the group B (17 females, 15 males; 17 Kotlow’s class III, 15 Kotlow’s class IV) had undergone a laser frenectomy. Both the procedures have been performed by the same operator for all the patients at the Oral Surgery Unit of Policlinico Universitario Agostino Gemelli (Rome, Italy) between May and July 2021. The data assessed during and after the operation were recorded in the clinical chart and retrospectively analyzed. Because of the retrospective nature of the present study, it was granted an exemption in writing by the local ethics committee. For the realization of this study, the guidelines present in the Declaration of Helsinki have been read and followed. The selection of the study population was based on the following criteria:

- Subjects had to be aged between 8 and 12 years old, in good general health conditions and with no hematic problems;
- Subjects could not be under orthodontic treatment;
- Subjects could not present genetic or congenital pathologies;
- Subjects who need to take drugs were excluded;
- Subjects presenting viral diseases were excluded;
- The parents of the subjects had to sign an Informed Consent in order for the subject to join the study.

### 2.2. Preoperative Measurements and Treatment

At baseline, all subjects were given a questionnaire in which they would report the degree of pain perceived immediately after the end of the anesthesia, measured with the Numeric Rating Scale (NRS), with 0 indicating the absence of pain and with 10 the maximum pain perceived pain in life. The same questionnaire was given to them again a week after the operation and the pain perceived at that moment was recorded. In additional, the clinical parameters that make up the EHS [36] (clinical signs of re-epithelization or CSR; clinical signs of hemostasis or CSH and clinical signs of inflammation or CSI (Table 1) were measured in a follow-up visit 24 h after the operation and used to evaluate the quality of the healing of the wound in each group. Tongue training exercises were prescribed to everyone after surgery; all patients took paracetamol on the evening of the surgery, before going to bed.

### 2.3. Operative Protocol for Group A

**Figure 1:**
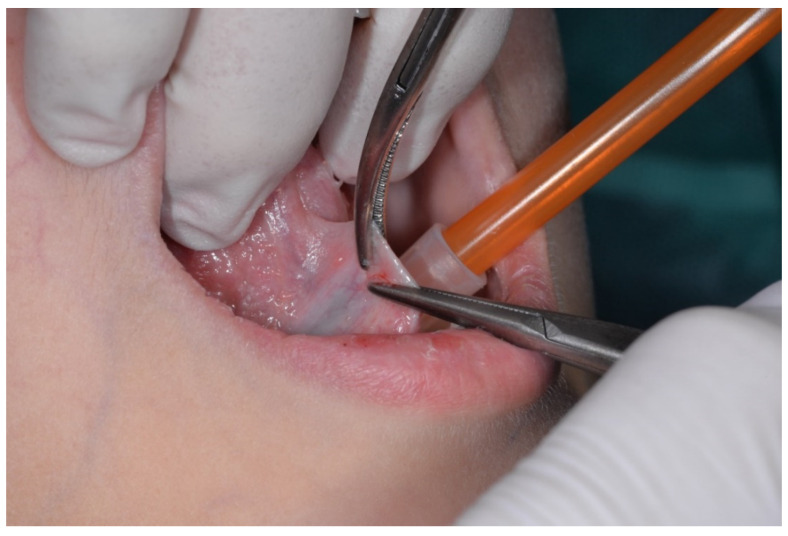
Clamping of the frenulum using two hemostatic forceps.

To block the transmission of pain, the surgeon performed an infiltration with mepivacaine 1:100,000, so that the lingual nerves that interest this region of the tongue were isolated. Moreover, this prevented the swelling of the site, in order to perform the procedure in the best possible conditions. Two hemostatic forceps were used to clamp the frenulum (one close to its insertion on the tongue and one close to the mouth floor) (Figure 1). Since this is a very sensible area for the presence of the Wharton’s ducts close to the insertion of the frenulum to the mouth floor, the forceps were used very carefully.

**Figure 2:**
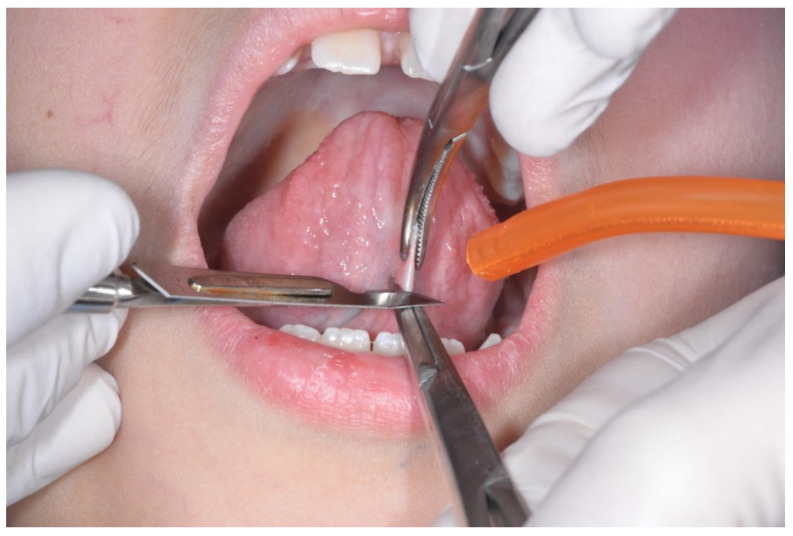
Starting the surgery using a 15c blade.

The surgeon used a scalpel with a n15c blade to perform the section of the frenulum (Figure 2).

**Figure 3:**
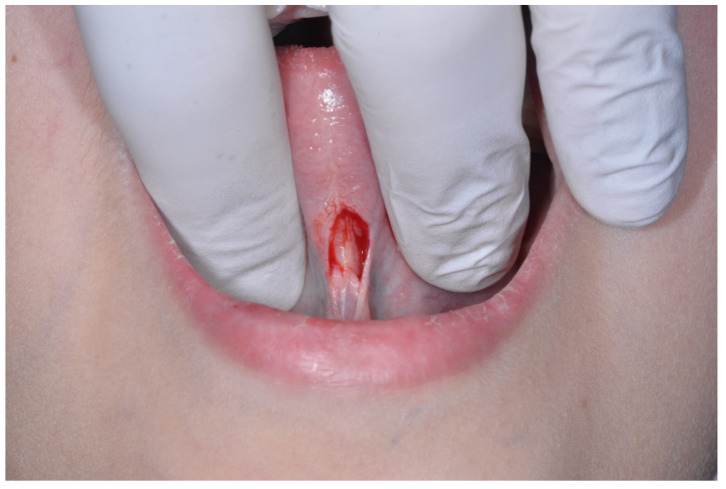
Evidencing the diamond-shaped pattern of the incision.

In all the cases the incision left a diamond-shaped pattern in the mucosa that has been extended laterally, to guarantee the movements of the tongue (Figure 3).

**Figure 4:**
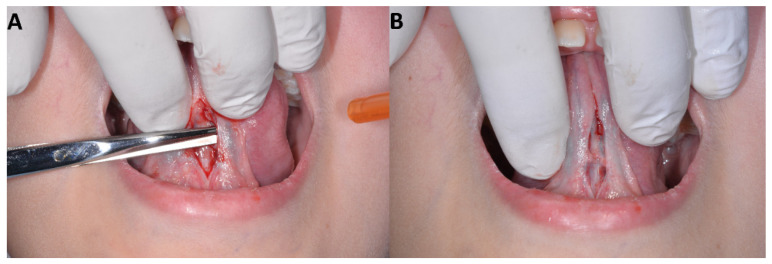
The mucous layer being separated from the underlying muscle layers using a scissor (**A**) and the increased mobility obtained throughout this operation (**B**).

The bleeding was controlled using the Metzebaum’s scissors, and the mucous layer was separated using a scissor from the underlying muscle layers, to make sure there would be no tension on the suture (Figure 4).

**Figure 5:**
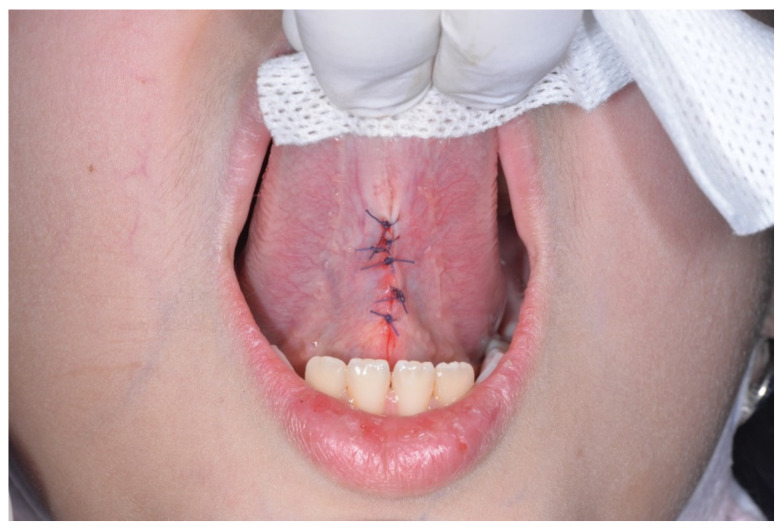
Result after suturing.

The surgeon used polysorb 4/0 wire to place from 3 to 6 interrupted sutures [37] (Figure 5). In no case it was necessary to fix postoperative complications, thanks to the punctilious observance of the procedure.

In all the cases, a treatment with paracetamol during the first 24 h (300 mg every 3 h), rinses with chlorhexidine mouthwash (3/4 a day) for a week, antibiotics therapy to prevent superinfections and a soft and cold diet for the first two days were prescribed. The sutures were removed after 10 days, but in 9 patients (31%) it was necessary to wait for two weeks, since the wound still had slight bleeding after 10 days.

### 2.4. Operative Protocol for Group B

**Figure 6:**
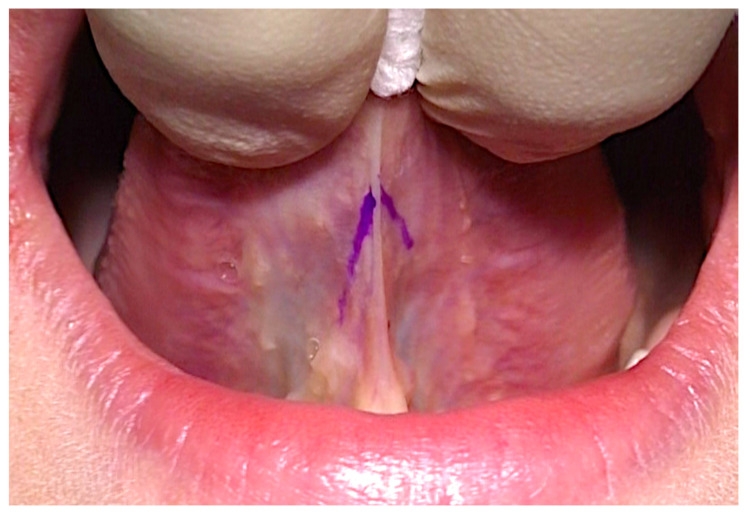
Positioning of the tongue and design of the incision line.

To block the transmission of pain, the surgeon performed an infiltration with mepivacaine 1:100,000. With the help of a surgical gauze, the patient’s tongue was gently pulled, interposing the frenulum between the index and middle fingers in order to prevent the deformation and the tension of the frenulum and the shape of the incision was drawn using a colored tip (Figure 6).

**Figure 7:**
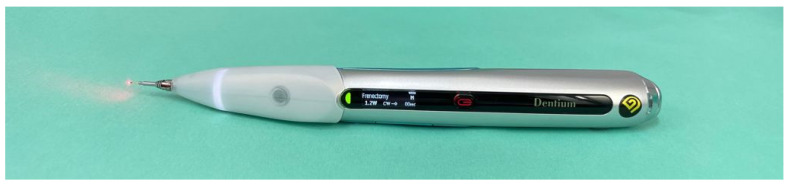
Laser settings.

The procedure was performed using a diode laser (K2 mobile series laser, Dentium, Korea), with a wavelength equal to 980 nm, 330 mJ × 50 Hz frequency, average power of 1.2 watts and pulse interval of 1 ms in direct contact with the tissues to be treated (Figure 7).

**Figure 8:**
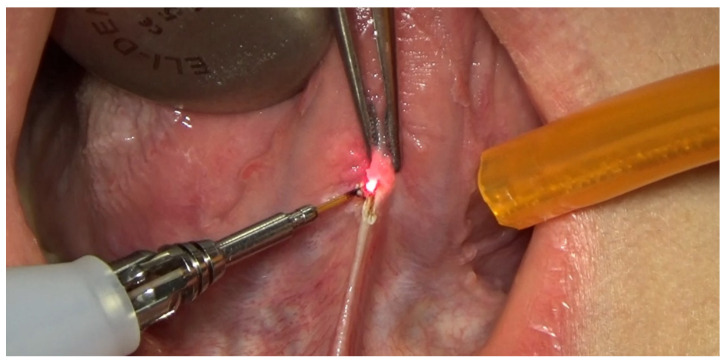
Surgery being performed.

With activated fiber, the operator started cutting the frenulum in the insertion area closest to the tip of the tongue and proceeded distally to the oral floor, parallel to the long axis of the tongue. The cut had to be performed on both the right and left sides, ideally tracing an inverted “V”-shape, with laser tip in contact mode, using brushing movements. This procedure caused a progressive release of tractions that was further increased by deepening the cut towards the midline, allowing an agile lifting of the tongue towards the palate. In order to allow the rapid and precise execution of the procedure, it was necessary to activate the fiber every time the cutting power was inhibited, also combining the removal of residues using a gauze moistened with physiological solution. The entire procedure has to be performed without irrigation (Figure 8).

**Figure 9:**
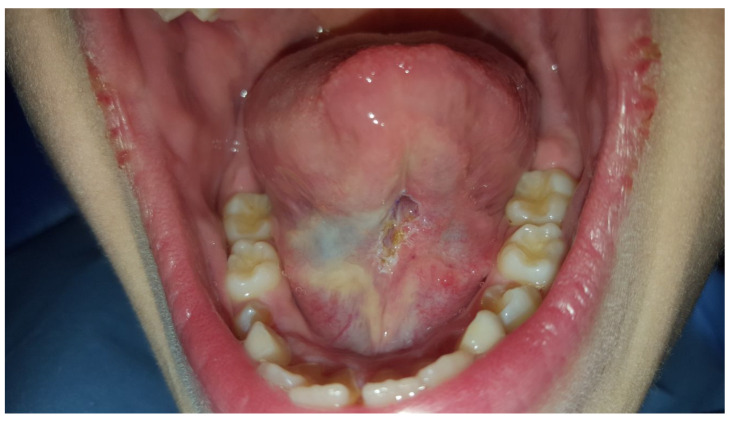
The situation immediately after surgery. It is possible to observe the lack of bleeding and the absence of edema.

There was no bleeding, because of the fact that the mucogingival and connective tissues are coagulated before and, only then, cut by the laser (Figure 9).

**Figure 10:**
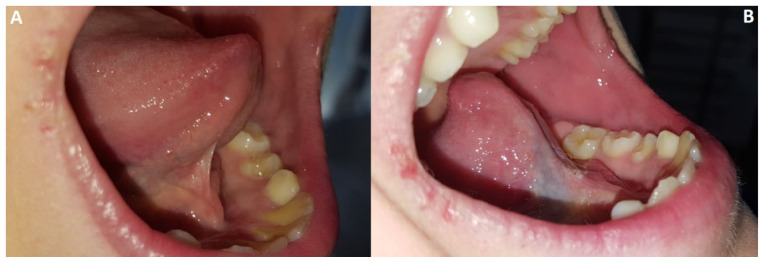
The mobility of the tongue is extremely limited before the operation (**A**) while it is visibly increased already in the immediate post-operative (**B**).

The section of the fibers was performed to enhance the mobility of the tongue. After the procedure it was possible to use the laser fiber in defocused mode, in order to bio-stimulate the tissues surrounding the treated area and reduce postoperative edema and pain. There was no need to administer antibiotics and/or anti-inflammatory drugs [38]. No sutures were needed, since the laser was able to cauterize the tissue, preventing bleeding. Immediately after the surgical procedure, all the patients had a clear perception of the ease in tongue movement and lifting, and it was possible to observe an objective improvement in the level of the mobility of the tongue (Figure 10).

In no case has edema or post-operative complications been registered.

### 2.5. Statistical Analysis 

Values were expressed as mean and SD for continuous variables or absolute frequency and percentage for categorical variables. Continuous variables were compared with Student’s T-test. All statistical analyses were carried out using STATA14 for windows software with a two tailed p value of 0.05 used as a threshold for significance.

## 3. Results

**Figure 11:**
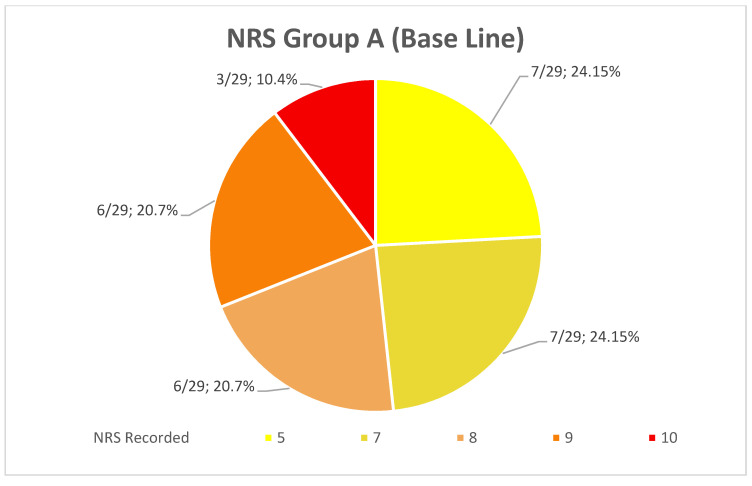
Distribution (numerical and percentage) of the NRS recorded in patients of Group A immediately after surgery.

**Figure 12:**
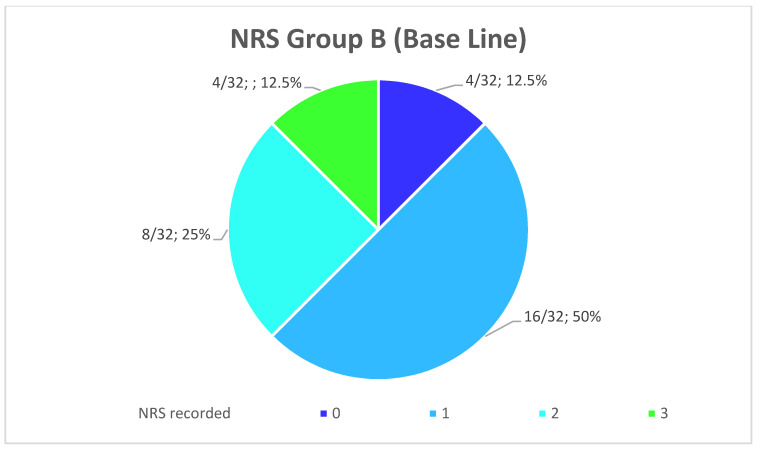
Distribution (numerical and percentage) of the NRS recorded in patients of Group B immediately after surgery.

The average NRS to indicates the level of pain the patients felt immediately after the end of the anesthesia in the patients of Group A was 7.44 ± 1.68 going from 5 to 10 (Figure 11). Instead, the average NRS in the patients of Group B in the same moment was 1.38 ± 0.8 going from 0 to 3 (Figure 12).

**Figure 13:**
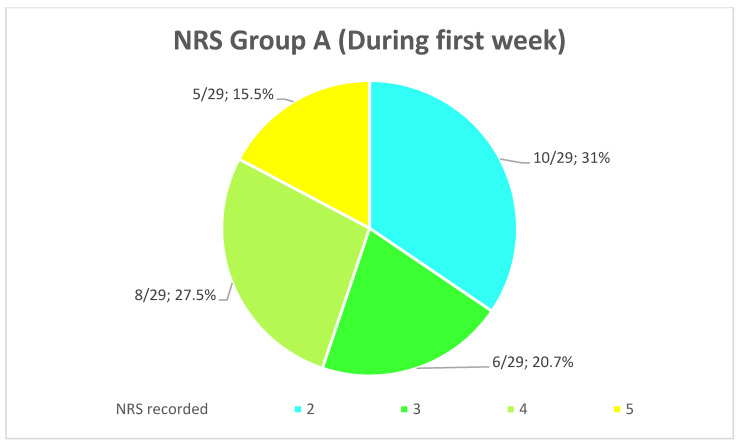
Distribution (numerical and percentage) of the NRS recorded in patients of Group A the first week after surgery.

**Figure 14:**
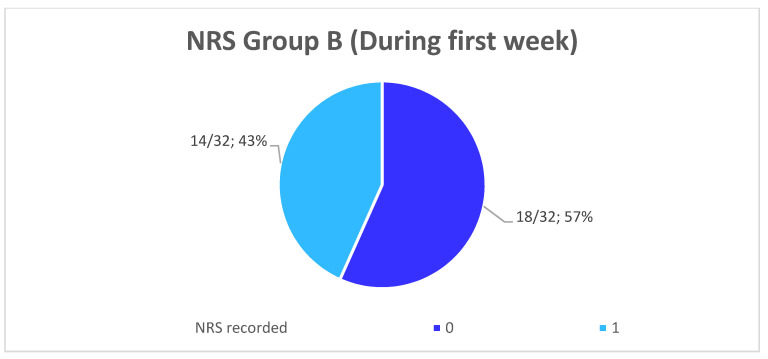
Distribution (numerical and percentage) of the NRS recorded in patients of Group B during the first week after surgery.

**Figure 15:**
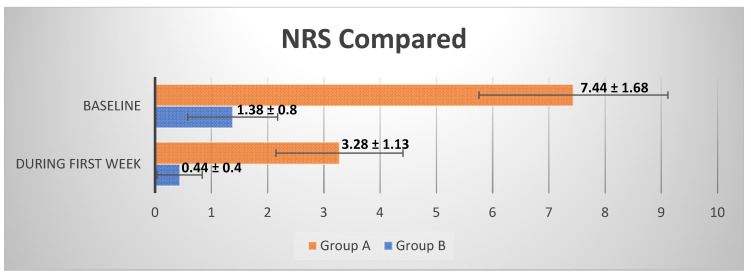
Comparison between the average NRS ± SD recorded at baseline and during the first week after surgery in Groups A and B.

The comparison of the average values between the groups showed a reduction of 80.6% (*p* < 0.001) of the NRS recorded immediately after the operation in Group B compared to Group A. Furthermore, the patients of Group A are more likely to undergo a slower and more uncomfortable healing, confirmed by their NRS recorded the first week after surgery, with an average score of 3.28 ± 1.13 going from 2 to 5 (Figure 13), while the patients of the Group B presents an average NRS score during the first week of 0.44 ± 0.4 going from 0 to 1 (Figure 14), so that a reduction of 86.58% (*p* < 0.001) of the NRS recorded the first week after the operation in Group B compared to Group A was observed (Figure 15).

**Figure 16:**
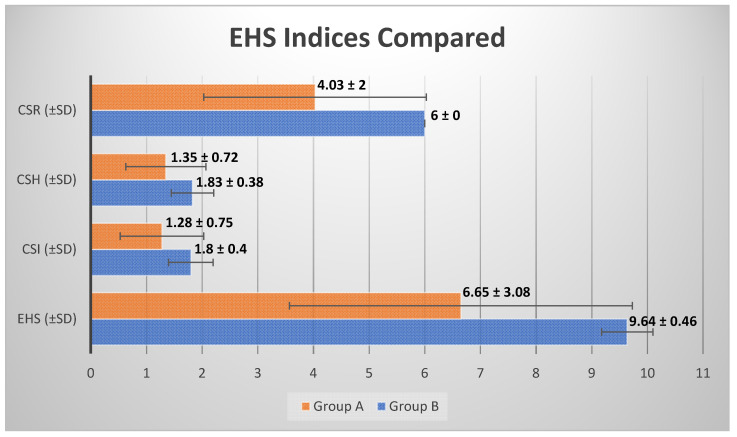
Comparison between the average EHS indices ± SD in Groups A and B.

The average EHS to assessing the quality of healing of the wound 24 h after the operation in the patients of Group A was 6.65 ± 3.08 going from 0 to 10 (Table 2), while the average EHS in the patients of Group B was 9.64 ± 0.46 going from 8 to 10 (Table 3), highlighting a mean difference in the average EHS between the two groups of 2.99 (45%), with a quality of the healing within the first 24 h significantly increased (*p* < 0.001) in patients of group B (Figure 16 and Table 4).

It was observed that in seven cases (24.15%) of Group A, a 24 h edema occurred, and treatment with NSAIDs or corticosteroids was necessary. Furthermore, for the patients of Group A, the presence of intraoperative bleeding was recorded while for the patients of the Group B there was no intraoperative bleeding improving the view of the surgical site. Lastly, the average operation time in patients of Group A was 41 ± 12 min, lasting from 29 to 52 min, while the average operation time in patients of Group B was 18 ± 5 min, lasting from 13 to 23 min, showing a significant reduction in the intraoperatory time required (*p* < 0.001).

## 4. Discussion

There are specific clinical criteria by which the diagnosis of ankyloglossia can be formulated [1]: if the patient is asked to open his mouth it will be impossible for him to touch the palate with the tip of his tongue; mechanical bifidity of the tongue or presence of a median furrow in protrusion; the patient presents no space or very little space under the tongue; and if the patient is asked to completely extrude the tongue it will be impossible for him to go beyond the vermilion lip, causing a bending of the central body of the tongue. Even though is preferable to treat this disease at a very early age, in order to avoid problems in the capacity to speak and orthodontic problems like lingual inclination of the lower incisors, diastemas, anterior bean, and dental rotation [7,8,10,11,12,13], it is also possible to perform these procedures in adult patients presenting, together with ankyloglossia, dysphonia problems [7,8,10,11,12,13], nodules on vocal cords, snoring problems, jaw disharmony, or swallowed-postural syndrome [1]. It is also recommended to ensure that a short frenulum does not cause instability in a plane of mobile prothesis rehabilitation, in which case surgery is strongly recommended, since these procedures can be particularly hard to perform in patients presenting complete form of ankyloglossia.

While the conventional surgical method can present multiple complications depending or not on the patient, such as excessive bleeding, prolonged healing times, excessive discomfort of the patient, appearance of keloids and others, according to the results, diode laser presents a series of advantages over the conventional surgical technique. Those advantages are due to the fact that the laser technique has a completely different operating principle with respect to classic surgery technique, based on the interaction with tissues. In fact, the laser does not just “cut” the structures that compose the frenulum (collagen and elastic fiber), but it causes a denaturation and, at 60 °C, tissue coagulation [39]. In fact, the scalpel causes the removal of the edges of the fibrous structures and the elimination of the tension, but maintains both the sections intact, causing the reaction of the body to try to repair the damage, causing inflammation [40]. Instead, the denaturation caused by the laser beam causes, first, a significant removal of the fibrous heads caused both from the reduction in the physiological traction and the elimination of a fibers; a decrease in the faint chemical hydrogen bonds between the collagen trope chains and, moreover, of the stronger bonds between the amino acids that arrange the protein structures (like lysine); this brings a lowering of the distance among the amino acids themselves with clotting fall [41]. It is clear now that this procedure brings a more difficult repairing process for fibroblasts, which is not immediately feasible in the coagulated sites, and is now considerably delayed. This is related to the fact that compared to the classical surgical technique, with the laser frenectomy a larger area is involved in the coagulation process and a major quantity of tissue is removed, so that the coagulated heads must be absorbed first. Secondly, new fibers need to be deposited, the two heads must recover the tension, and in the end, the previously sectioned fiber reshaped to the right length. This is a very long and complicated process that further delays the repairing and inflammation process [42]. Moreover, the heads of the fibers present coagulation and damage to their internal structures, as well as increased distance between them because of the wrinkling of the collagen fiber due to the increased temperature, causing a bigger hiatus between the two heads of the sectioned fibers compared to the one caused by the scalpel, which is microscopic and leads to an immediate repairing process [43]. All of this leads to a reduction in postoperative inflammatory exudate in situ, swelling, and pain. Moreover, it leads to a faster execution of frenectomy, the total absence of superinfection after surgery and, thus, the absence of antibiotic prescription, the slight wound contraction, and the lack of residual scarring. Moreover, using the laser in pulsed mode can guarantee all the above-mentioned specific effects and, also, ensure the non-over-heating of the tissue. In this way, the tissue is prevented from being heated more than is required, avoiding damaging the surrounding anatomical structures. Last but not least, is the fact that laser frenectomy can be performed without the use of a scalpel, which lessens bleeding and the need for sutures. Laser frenectomy can be performed in less time with very little discomfort for patients, so that even children are more apt to undergo surgery, therefore making it easier for patients to repeat the treatment in case of relapse, because of the absence of a previous traumatic experience.

Similar outcomes have been highlighted in other recent studies [44,45,46,47,48,49,50,51,52,53], making laser-assisted frenectomy a more valuable option compared to the conventional surgical method used in the last years. In particular, Nammour et al. [50] focused on the reduced need for sutures, while Brignardello-Petersen [48] and Viet et al. [53] highlighted different positive outcomes in diode laser frenectomy when compared to the traditional surgical method, such as reduced discomfort of the patients, reduction in the operation time, and reduced need of infiltration anesthesia. 

## 5. Limits of the Study

The main limitation of this study is certainly represented by its retrospective nature, due to which biases could have occurred due, for example, to the impossibility of dividing the two groups equally, taking in consideration ethnicity, level of oral hygiene, socio-economic situation, or other variables that could have been included in a prospective study. Furthermore, it was necessary to take in consideration only the data that are routinely collected, limiting the variables that were possible to analyze, while a prospective study would have made it possible to study more specific variables, such as the variation in the mobility of the tongue, as well as of the ability to speak, chew, and swallow.

Another limitation is the limited number of patients that was possible to include in the study, which does not guarantee a sufficient sample to consider the result obtained valid on a large scale. It would be necessary to conduct a similar study involving a greater number of patients in order to confirm that the results obtained are valid even in presence of a greater number of variables.

Furthermore, the measurement system used to define the pain felt by patients after the operation and during the first week is a numerical index (NRS), which presents the bias to influence patients in the evaluation of their condition, as they tend to refer to the values placed at the extremes of the scale, in a positive or negative sense. It would have been more appropriate, in order to make the measurement more objective, to use a scale of the VAS (Visual analog scale) type, which allows patients to indicate an evaluation based on drawings or colors, without being influenced by numerical measurements.

## 6. Conclusions

Within the limits of this study, results suggest that laser frenectomy could be considered as a valid option to the conventional surgical method, particularly in patients with problems that prevent them from undergoing interventions of longer duration and patients who are particularly sensitive to pain. The results are encouraging and justify further studies that could include a larger number of subjects, in order to confirm on large-scale, the results obtained.

## Figures and Tables

**Table 1:** EHS: Early Wound Healing Score, CSR: clinical signs of re-epithelialization, CSH: clinical signs of hemostasis, CSI: clinical signs of inflammation.

Parameter	Description	Points
CSR	Merged incision margins	6
	Incision margins in contact	3
	Visible distance between incision margins	0
CSH	Absence of fibrin on the incision margins	2
	Presence of fibrin on the incision margins	1
	Bleeding at the incision margins	0
CSI	Absence of redness along the incision length	2
	Redness involving <50% of the incision length	1
	Redness involving >50% of the incision length and/or pronounced swelling	0
Maximum total score: 10		

**Table 2:** Clinically recorded EHS parameters for patients of Group A.

Patient	Age	Sex	Kotlow’s Class	Length of the Operation (Minutes)	CSR	CSH	CSI	EHS
1	8	F	III	38	6	2	2	10
2	9	F	IV	36	6	2	1	9
3	9	F	III	41	6	1	2	9
4	10	M	IV	45	3	1	1	5
5	10	M	III	47	3	0	1	4
6	9	M	III	48	6	2	1	9
7	11	F	IV	36	0	0	0	0
8	11	M	IV	38	6	1	1	8
9	8	F	III	34	3	1	0	4
10	10	F	III	39	6	2	2	10
11	9	M	IV	37	6	1	2	9
12	10	F	III	39	3	2	1	6
13	9	F	IV	47	3	1	2	6
14	10	F	IV	44	6	2	1	9
15	11	M	III	49	0	0	0	0
16	12	M	III	52	0	0	0	0
17	9	F	IV	39	3	1	0	4
18	9	M	III	37	3	1	2	6
19	12	M	IV	46	6	2	2	10
20	8	F	III	29	3	2	2	7
21	9	M	III	37	6	2	2	10
22	12	F	IV	41	3	1	1	5
23	8	F	III	33	3	2	1	6
24	10	M	IV	43	3	2	2	7
25	9	M	III	46	6	1	2	9
26	10	M	III	47	3	1	1	5
27	11	F	IV	41	6	2	2	10
28	8	F	III	40	6	2	2	10
29	12	M	IV	38	3	2	1	6

**Table 3:** Clinically recorded EHS parameters for patients of Group B.

Patient	Age	Sex	Kotlow’s Class	Length of the Operation (Minutes)	CSR	CSH	CSI	EHS
1	9	F	IV	16	6	2	2	10
2	9	F	III	19	6	2	2	10
3	10	M	III	14	6	2	2	10
4	8	M	IV	19	6	2	2	10
5	12	F	III	18	6	2	2	10
6	12	M	IV	18	6	1	1	8
7	10	F	III	13	6	2	2	10
8	11	F	III	17	6	2	2	10
9	9	F	IV	16	6	2	2	10
10	9	M	IV	19	6	2	1	9
11	8	M	III	18	6	1	2	9
12	10	F	IV	22	6	2	2	10
13	9	F	III	18	6	2	2	10
14	11	M	III	17	6	2	2	10
15	11	F	IV	19	6	2	2	10
16	9	M	IV	23	6	2	2	10
17	8	M	III	21	6	1	2	9
18	9	F	III	13	6	2	2	10
19	10	M	IV	15	6	2	1	9
20	11	M	III	17	6	2	1	9
21	10	F	IV	21	6	2	2	10
22	12	F	III	17	6	2	2	10
23	12	F	IV	20	6	2	2	10
24	10	M	III	23	6	1	1	8
25	9	M	IV	18	6	2	2	10
26	9	F	III	15	6	2	2	10
27	10	F	IV	17	6	2	2	10
28	9	M	III	18	6	2	1	9
29	8	M	III	21	6	2	2	10
30	8	M	IV	14	6	1	1	8
31	12	F	III	17	6	2	2	10
32	11	F	IV	18	6	2	2	10

**Table 4:** Comparison of all the variables considered between Group A and B, with SD and *p*-Value.

Variable	Group A	Group B	*p*-Value
Gender (M/F)	14/15	15/17	0.91
Age (±SD)	10 ± 1.28	9.8 ± 1.3	0.73
Kotlow’s class (III/IV)	16/13	17/15	0.87
Time of the operation (min) (±SD)	41 ± 12	18 ± 5	<0.001
NRS (Baseline) (±SD)	7.44 ± 1.68	1.38 ± 0.8	<0.001
NRS (First week) (±SD)	3.28 ± 1.13	0.44 ± 0.4	<0.001
CSR (±SD)	4.03 ± 2	6 ± 0	<0.001
CSH (±SD)	1.35 ± 0.72	1.83 ± 0.38	0.0018
CSI (±SD)	1.28 ± 0.75	1.8 ± 0.4	0.0014
EHS (±SD)	6.65 ± 3.08	9.64 ± 0.46	<0.001

## Data Availability

The data presented in this study are available on request from the corresponding author. The data are not publicly available due to privacy reasons.
